# An evaluation of temporal and club angle parameters during golf swings using low cost video analyses packages

**DOI:** 10.1038/s41598-022-17175-2

**Published:** 2022-08-17

**Authors:** Henry H. Hunter, Ukadike C. Ugbolue, Graeme G. Sorbie, Wing-Kai Lam, Fergal M. Grace, Antonio Dello Iacono, Minjun Liang, Frédéric Dutheil, Yaodong Gu, Julien S. Baker

**Affiliations:** 1 Faculty of Sports Science, Ningbo University, Ningbo, China; 2 Division of Sport and Exercise, School of Health and Life Sciences, University of the West of Scotland, South Lanarkshire, Glasgow, G72 0LH UK; 3 Division of Sport and Exercise Sciences, Abertay University, Dundee, DD1 1HG UK; 4 Future Regions Research Centre, Federation University Australia, Mt Helen, Ballarat, Victoria 3353 Australia; 5 Li Ning Sports Science Research Center, Li Ning (China) Sports Goods Co. Ltd, Beijing, China; 6 Department of Kinesiology, Shenyang Sports Institute, Shenyang, China; 7 CNRS, LaPSCo, Physiological and Psychosocial Stress, University Hospital of Clermont-Ferrand, CHU Clermont-Ferrand, Preventive and Occupational Medicine, WittyFit, Université Clermont Auvergne, Clermont-Ferrand, France; 8 Centre for Health and Exercise Science Research, Department of Sport, Physical Education and Health, Hong Kong Baptist University, Kowloon Tong, Hong Kong; 9 Biomechanics Laboratory, Division of Sport and Exercise, School of Health and Life Sciences, University of the West of Scotland, South Lanarkshire, Glasgow, G72 0LH UK

**Keywords:** Health care, Engineering, Mathematics and computing, Physics

## Abstract

The purpose of this study was to compare swing time and golf club angle parameters during golf swings using three, two dimensional (2D) low cost, Augmented-Video-based-Portable-Systems (AVPS) (Kinovea, SiliconCoach Pro, SiliconCoach Live). Twelve right-handed golfers performed three golf swings whilst being recorded by a high-speed 2D video camera. Footage was then analysed using AVPS-software and the results compared using both descriptive and inferential statistics. There were no significant differences for swing time and the golf phase measurements between the 2D and 3D software comparisons. In general, the results showed a high Intra class Correlation Coefficient (ICC > 0.929) and Cronbach’s Coefficient Alpha (CCA > 0.924) reliability for both the kinematic and temporal parameters. The inter-rater reliability test for the swing time and kinematic golf phase measurements on average were strong. Irrespective of the AVPS software investigated, the cost effective AVPS can produce reliable output measures that benefit golf analyses.

## Introduction

Historically, kinesiological analyses of performances have been evaluated in both clinical^1–3^ and sport^4^ environments using low cost two-dimensional (2D) and expensive three-dimensional (3D) photogrammetry kits. It is well known that 3D motion capture is regarded as the gold standard for human movement analyses^5,6^. However, it is expensive and imposes financial costs affordable by very few researchers and coaches. With the advent of digital and mobile technology, clinical-based, laboratory-based and field-based kinesiological data can be captured, processed and analysed using bespoke 2D Augmented-Video-based-Portable-Systems (AVPS).

Ugbolue and colleagues have captured and evaluated clinical datasets using both 2D and 3D motion systems^1,7^. This has been supported by previous studies on 3D sport related projects in the area of golf swing biomechanics^8,9^ and 2D golf swing related projects^10^. 2D motion systems are the next best alternative when 3D optical systems, inertial systems and electromagnetic systems which are widely used in golf are unavailable. While few sport related field events can be 3D motion assessed within the laboratory there is still need from a practical, training, rehabilitation and coaching perspective to be able to understand the mechanics and techniques associated with performance using advanced 2D motion systems. These concerns suggest the need for effective 2D motion system sport related alternatives that can capture slow and fast-paced dynamic and complex multiple planar motions. The introduction of technology in sports training and coaching remains impactful and globally continues to play a significant role in the development of sport^11^. The versatility of 2D video technology in sport using high-speed video has previously been investigated^12–14^ and remains an inexpensive solution for motion analysis that is relevant in everyday elite coaching and training sessions.

Several software products are available to analyse the 2D output from an AVPS. More products that are popular include SiliconCoach Pro (SiliconCoach Pro version 8.0.7.2. computer software 2019; https://www.siliconcoach.com/siliconcoachPro), SiliconCoach Live (SiliconCoach Live version iOS (via App Store for iPhone and iPad); https://www.siliconcoach.com/SiliconcoachLIVE).and Kinovea (Kinovea version 0.8.27. computer software 2018; https://www.kinovea.org/). All three software products have made a significant impact within the fields of clinical practice, education, sports and indeed, as assessment tools with strong applications within research and performance analyses.

SiliconCoach Pro is an effective tool designed to improve an athlete’s performance. Performances can be analysed in detail using high quality video, flexible layouts and easy work flow features^15^. SiliconCoach Live also has similar features where videos can be analysed and feedback provided in a cloud based environment^16^. In many cases these 2D software systems are used not only for simple gait analysis but also for analysis of many sports related movements especially golf. Test–retest reliability studies^17,18^ have been conducted using the SiliconCoach software but are yet to be fully explored in terms of validating outcome measures associated with the golf swing. 2D software such as SiliconCoach has been used to assess the dynamic range of motion of the knee joint^17^. This study concluded that SiliconCoach was beneficial in the determination of the end range of static and dynamic motion, and valuable from a functional and cost-effective perspective especially within the clinical environment. From a sporting context a further laboratory-based study focused on developing a method to measure core ability among healthy female gymnasts^18^. The study confirmed that using SiliconCoach provided an accurate and reliable approach to assessing core ability exercise performance.

Kinovea is a completely free and open source video annotation tool with applications for education, rehabilitation and sport^19^. The Kinovea software has undergone validity and reliability assessments^20–25^. These assessments have benefitted interpretations of clinical^21,26–31^ and sport performance^22,23,32,33^ evaluations using a cost effective valid and reliable tool. Despite the Kinovea software having applications within clinical and sport related settings, from a research standpoint no studies have evaluated the efficacy of the software in terms of outcome measures associated with golf biomechanics and performance.

Comparable AVPS devices have also been deemed acceptable for use within clinical settings as they produce similar results to that of the motion capture system. This is an area of importance because it will allow smaller clinics to use the AVPS as a viable replacement to the more expensive system, allowing rehabilitation costs to be reduced for the same results leading to less financial expenditure to both the patient and the clinics’ running costs^34^. Although 2D AVPS are acceptable within the clinical and sports settings, many of the accompanying software products appear to provide inaccuracies in their temporal and spatial output measures. These inaccuracies predominantly are initiated and driven by the users’ inability to identify the correct frames for measurement and the implementation of the accurate application of the software tools. Furthermore, these inaccuracies may lead to temporal and spatial variations and errors in the output measures^35,36^. Therefore, there is a need to evaluate popular software products that are used by clinicians, coaches and bio mechanists during their routine 2D video assessments. This knowledge gap raises questions as it is unclear whether significant variations exists between software products relating to the output measures reported.

There is a highlighted area of importance throughout the literature, which states that the need for rehabilitation clinics is increasing^37^. Previous research has identified that the AVPS are accurate within its own surroundings but may present problems for rehabilitation purposes when compared against the gold standard systems^1,2,7^. The AVPS can also become more important during the analysis of golf swings as it can help to identify potential injury risks. Even though the golf swing is not particularly intensive or exhausting, it still stresses the skeletal-muscle systems, which can be related to golf injuries including neck, shoulder and back pain^38–40^. These injuries potentially could influence changes in the movement patterns, lumbar spinal loading and muscle activity in relation to the mechanical movement of the golf swing^41^.

Despite thousands of articles dealing with analysis of golf performance, it has been identified that the game of golf remains physically and mentally complex^42^. Biomechanical analysis of a golf swing is widely known as being difficult to interpret due to the complexity of the swing, as it has a 3D motion, multi-planar sequence, which is performed at great speed. There are numerous kinetics and kinematics variables that can be explored when analysing a golf swing to understand its mechanical complexity^43^. The main 2D aspects of a golf swing can be broken down into several segments, for example, how a player stands at address, weight shift during backswing and acceleration, wrist hinge angles, club release angles and torques of the shoulders and wrists throughout the swing. These aspects all contribute to the performance of a golf swing^44^. Furthermore, Kwon and associates suggested that due to the golf swing being a complex skill of motion analysis, advanced methods of analysis are required to determine unique aspects of the skill. These advanced analysis methods include, detecting the exact instant of impact, definition of the athlete’s body position and the golf club in various frames and the determination of different planes of the club during the swing^45^.

Although performance-based outputs such as club head speed, ball, speed, club path and launch angle are not evaluated in this study, emphasis will be channelled towards other kinematically driven performance outcome measures that utilise the software drawing tools and time software features. Therefore, the shaft angle or golf club inclination angle (°) will be calculated at each of the five golf phases (address, top of backswing, acceleration, impact and follow through) together with the total golf swing phase time (swing time). In golf coaching there are specific times in the swing when the shaft angle alone is of interest. In the frontal plane that is mainly at impact and the top of the swing. In the sagittal plane, it would be a mid-backswing (shaft vertical from face on), or mid-downswing. Timing in the golf swing is of interest when comparing the time of the backswing versus the time of the downswing. This provides some sense of tempo.

While some discrepancies in measurement techniques may have consequential effects on temporal and spatial output measures, from a software user perspective, clarity and reassurances are needed to show the viability and robustness of the 2D AVPS in the context of temporal measurements and shaft angle with respect to the Global Coordinate System. It is evident that commercial applications of motion capture in golf using non-optical systems (e.g. inertial systems, magnetic systems) and optical systems (e.g. marker less optical systems, active optical systems, passive optical systems) have been reported^46^. Though most of the research studies using planar kinematics may be considered dated^47–51^, these classical studies still have useful technical and methodological applications that are of benefit in analysing 2D golf datasets. To date there are no recent research studies to support the use of 2D AVPS in the analysis of the golf swing. From a technical, methodological and applied technology perspective, this study reinstates the importance and value of golf related temporal and spatial outcome measures derived from using less complex technology have in today’s world. Therefore, the purpose of this study was to evaluate the efficacy of the AVPS as a useful assessment tool for measuring golf swing time and club angle parameters using three commercial popular software namely Kinovea, SiliconCoach Pro and SiliconCoach Live. We hypothesize that there will be no significant differences between the three commercial software packages; and no significant differences between temporal outputs from the commercial software and the software from the gold standard motion analysis system.

## Methods

### Participants

Twelve right-handed subjects (Male: 6, Age: 23.3 ± 4.3 years, Mass: 88.3 ± 16.6 kg, Height: 180.5 ± 4.4 cm; Female: 6, Age: 21.8 ± 1.7 years, Mass: 65.2 ± 5.7 kg, Height: 165.9 ± 8.1 cm) participated in this laboratory-based study. Informed consent was obtained from all participants. All participants completed a Physical Activity Readiness Questionnaire and consent form and were required to be healthy and injury free. In addition, all methods were carried out in accordance with relevant guidelines and regulations. No previous experience of playing golf was required (experience ranged from novice through to elite players). All experimental data collection was performed at the same time of day to minimize diurnal variation effects. There were five novice players (3 males and 2 females) without a handicap and seven experienced golfers (6 males and 1 female) with a Mean ± SD handicap of 18.0 ± 6.0. The ethics committee of the University of the West of Scotland approved the study (Approval Number: 5-3-14-002).

### Experimental design and protocol

A golf mat and driving net were placed in the centre of the biomechanics laboratory. One 2D camera was placed facing the frontal plane of the golfer to ensure that the full motion of the golf swing was captured. To ensure consistency throughout the recording of all trials, a visible mark was placed on the floor behind the golf mat to indicate the stance/starting position of each participant prior to data collection. Each participant performed their own warm up procedures prior to the test and when comfortable, performed three recorded golf swings. The 2D camera (EXILIM, Casio, USA) was mounted on a tripod at a height of 1 m, positioned at 4 m relative to the golfer and set to record at 1/2000 shutter speed and 240 Hz to allow for successful video capture during the high-speed movement.

The golf shots were performed using a Taylor made (Basingstoke, UK) Speed Blade Stiff Shaft 7-iron, with a shaft length of 37 in. The same Titleist Pro-V1 (Titleist, Cambridgeshire, UK) golf balls were also used for all golf shots. Four evenly spaced retroreflective markers were placed on the shaft of the 7-Iron Golf Club to identify the phases of the golf swing. The golf ball was covered with retroreflective material. Its position was standardised and not placed on a tee. Other retroreflective markers were placed on the segments and joints of the lower limb of the golfers following the Plug-in-Gait model (https://docs.vicon.com/).

SiliconCoach Pro (SiliconCoach Pro version 8.0.7.2. computer software 2019; https://www.siliconcoach.com/siliconcoachPro), SiliconCoach Live (SiliconCoach Live version iOS (via App Store for iPhone and iPad); https://www.siliconcoach.com/SiliconcoachLIVE).and Kinovea (Kinovea version 0.8.27. computer software 2018; https://www.kinovea.org/).

**Figure 1:**
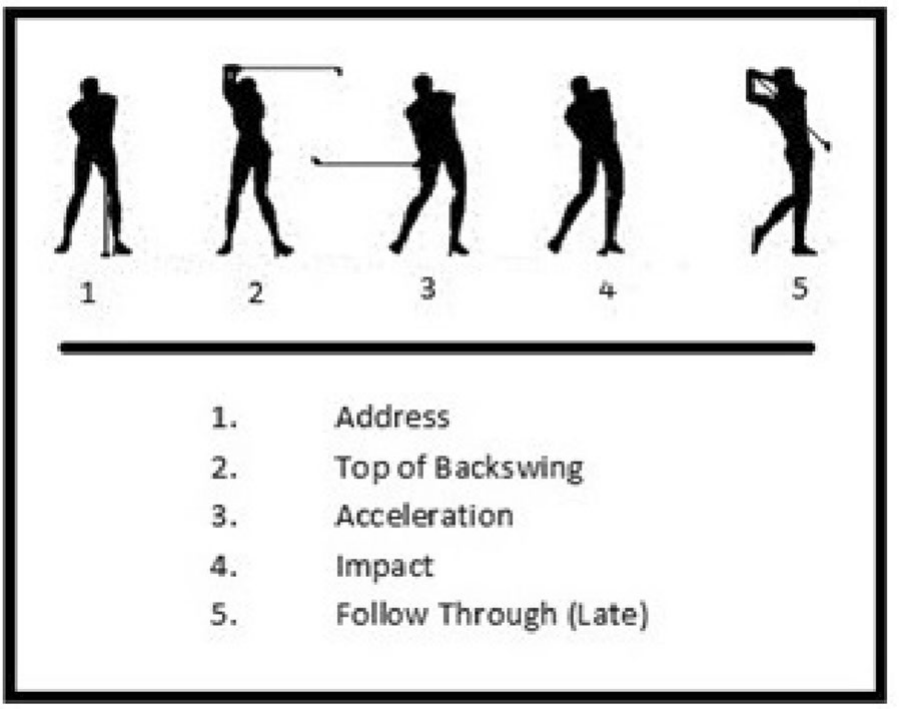
Silhouette illustration showing analysed phases of the golf swing.

All 2D video data was transferred from the camera to the laptop before being analysed with SiliconCoach Live (SiliconCoach Live version iOS via App Store for iPhone and iPad; http://www.siliconcoach.com/SiliconcoachLIVE), SiliconCoach Pro (SiliconCoach Pro version 8.0.7.2. computer software 2019; http://www.siliconcoach.com/siliconcoachPro) and Kinovea (Kinovea version 0.8.27. computer software 2018, http://www.kinovea.org) software for all participant’s golf swings. These three systems all have video playback features, drawing tools, which can interpret joint and club angles, zoom in and out features as well as markers to show where the data was analysed on the video timeline. The golf club inclination angle (°) was calculated at each of the five golf phases (address, top of backswing, acceleration, impact and follow through) (Fig. 1). The phases of the golf swing were defined as follows:

1. *Address*: The address phase is measured as the last frame before the clubhead starts moving away from the ball. The address stance position involved taking the golf club with the arms extended, back straight and knees flexed, so that the centre of the club face lines up with the ball.
2. *Top of backswing*: This position is attained when the club face is at its highest point. Here the golf club begins movement until the backward motion stops. This position also defines the start of the downswing.
3. *Acceleration*: Upon the initiation of the forward swing, the acceleration phase begins from the horizontal club position until the club face encounters the golf ball
4. *Impact:* This is the point at which the club face contacts the golf ball. The early follow through occurs upon impact with the golf ball until the golf club is horizontal to the ground (left side for right-handed golfers and right side for left-handed golfers) and the initiation of late follow through transition begins.
5. *Follow through (late)*: The late follow through starts from when the club is horizontal to the ground until the end of the motion where the swing ends.

Data were sampled at 240 Hz from an eight camera Vicon Nexus Motion Analysis System (Oxford Metrics Ltd, UK) mounted on scaffolding at 1.5 m and 2.3 m heights. The camera setup and configuration provided an optimal position for the cameras to capture and record the movement of the retroreflective markers during the golf swings. The Vicon Nexus Motion Analysis System (Oxford Metrics Ltd, UK) underwent a dynamic and static calibration prior to embarking on data collection. The software used for the analyses was the Vicon Nexus 2.8.1 Software.

The golf club inclination angle (°) was calculated as the club angle with respect to the vertical. Specifically, the golf club inclination angle (°) was obtained using the angle tool in the 2D commercial software. This involved selecting the first and fourth retroreflective markers on the shaft of the golf club with respect to the vertical as the third point. The total golf swing phase time (swing time) was also calculated for each of the software packages. The inclination angle on the 3D Vicon Motion System was calculated by recording the co-ordinates of the positions of the first and fourth retroreflective markers at the phase of the swing and with respect to the club when vertical before the application of a trigonometric function. Swing time was measured from the end of the address phase to the end of the follow through phase. Four raters independently defined the golf phases and digitized the markers across the software and participants.

### Statistical analysis

Normality of data distribution was verified using the Shapiro–Wilk test. The average of three trials was determined for all participants and expressed as a function of the golf phases of measurement using both descriptive and inferential statistics. Standard Error of Mean (SEM) was used to quantify the absolute consistency of the measurement^52^, while the Standard Error of Measurement (SM) estimated the amount of error in the test. Both the SEM and SM were calculated with respect to the swing time and each golf phase of measurement. Coefficient of determination (*r*^2^) and Pearson product moment correlation coefficients were used to assess linear relationships between the 2D and 3D displacement golf club inclination angle (°) measures in the frontal plane with respect to the phases of golf swing measurements. The strength of the correlation (*r*) was interpreted as poor (0 to 0.49), moderate (0.50 to 0.75), and strong (> 0.75)^53^. Repeated measures ANOVA with a post hoc main effects comparison using the Bonferroni correction was applied to establish (a) if there were any significant differences between the three commercial software instruments and (b) whether there were any significant differences between the temporal outputs from the commercial software and the Vicon Motion System gold standard. Reliability of the 2D and 3D output measures were assessed using the Intraclass Correlation Coefficient (ICC) and Cronbach’s Coefficient Alpha (CCA) analyses. An inter-rater reliability analysis was also performed to fully connect the study to the stated hypothesis and goals. The alpha level was adjusted to reflect the six pairwise comparisons of the commercial software and the Vicon Motion System with respect to each of the golf swing phases. Therefore, the statistical significance was denoted as *P* ≤ 0.0083.

To assess the agreement between the 2D commercial software and the 3D Vicon Motion Analysis gold standard system a Bland–Altman test was performed. A Bland–Altman plot displaying the relationship between the difference and the mean was produced^54^. Evidence of proportional bias and any points located outside the upper and lower 95% Confidence Interval (CI) was recorded. Prior to plotting the difference against the mean in the Bland–Altman plot a one sample t test was performed. Using the Statistical Package for the Social Sciences (SPSS 25.0; IBM, Corp, Armonk, NY) software, the test variable was inputted as difference and the test value was set to zero. The mean and standard deviation were used to calculate the 95% Confidence Interval upper and lower limits. The p value from the one sample t test was examined and a non-significant difference (P > 0.05) suggested progress could be made with the Bland–Altman plot. A linear regression was applied to further inform about any potential proportional biases. Upon running the analysis using the Statistical Package for the Social Sciences (SPSS 25.0; IBM, Corp, Armonk, NY) the centre of interest was the coefficient outputs particularly, the unstandardized coefficient beta value for the mean. This value needs to be close to zero and the significant output recorded. If the mean value was significant (*P* < 0.05) then the results would suggest that there is a proportional bias in the Bland–Altman plot. If the mean is not significant (*P* > 0.05) an assumption of no proportional bias in the results can be made.

## Results

All temporal and kinematic datasets from the 2D commercial and 3D Vicon Motion System were normally distributed. The descriptive statistics for the temporal and phase measurement parameters are displayed in Table 1. The swing times were very close as indicated by the sizes of the mean and standard deviation for each of the commercial software. The SEM for all three AVPS showed the same output of 0.11. However, the SM showed a small deviation across the three AVPS with Kinovea showing a 0.01 s reduction when compared to the SiliconCoach Pro and SiliconCoach Live packages. The swing time outputs were very similar to the gold standard (Vicon Nexus 2.8.1 Software). The repeated measures ANOVA results revealed no significant differences (*P* = 0.763, *F* = 0.099) for the within-subjects’ effects and no significant differences (*P* > 0.502) for each of the pairwise comparisons.

**Table 1:** Summary results for club angle with respect to vertical (°) and swing time (s).

Measurement	Commercial software product															
	Kinovea				SiliconCoach Pro				SiliconCoach Live				Vicon Nexus 2.8.1 software			
	Mean	SD	SEM	SM	Mean	SD	SEM	SM	Mean	SD	SEM	SM	Mean	SD	SEM	SM
Swing time (s)	1.95	0.37	0.11	0.14	1.97	0.38	0.11	0.15	1.99	0.38	0.11	0.15	1.97	0.33	0.10	0.15
Address (°)	6.70	1.91	0.55	0.44	6.24	1.51	0.43	0.34	6.78	1.90	0.55	0.43	6.57	1.70	0.49	0.40
Top of backswing (°)	103.83	28.58	8.25	3.22	102.82	29.00	8.37	3.27	102.59	28.90	8.34	3.26	103.08	28.47	8.22	3.25
Acceleration (°)	88.38	2.98	0.86	2.01	87.03	3.56	1.03	2.40	87.19	2.83	0.82	2.27	87.53	3.01	0.87	2.23
Impact (°)	7.22	1.93	0.56	0.87	7.34	1.27	0.37	0.58	7.40	1.87	0.54	1.36	7.32	1.60	0.46	0.94
Follow through (°)	92.96	45.52	13.14	19.26	93.23	44.67	12.89	18.90	91.68	45.35	13.09	20.28	92.62	45.14	13.03	19.48

No significant differences were observed for the repeated measures ANOVA results between the software and the phases of the golf swing at Address (*P* = 0.148, *F* = 2.232); Top of Backswing (*P* = 0.699, *F* = 0.169); Acceleration (*P* = 0.018, *F* = 5.718); Impact (*P* = 0.835, *F* = 0.175); and Follow Through (Late) (*P* = 0.281, *F* = 1.346). Table 2 displays the significance of the correlation between the commercial video systems and the Vicon motion system. Also presented are the coefficient of determination, Pearson’s correlation coefficient, and associated 95% confidence intervals between the 2D and 3D analysis systems.

**Table 2:** Coefficient of determination, Pearson’s correlation coefficient, and associated 95% confidence intervals between 2 and 3D analysis.

Product comparison	Phases of the golf swing																			
	Address				Top of backswing				Acceleration				Impact				Follow through (late)			
	r^2^	r	*P*	95% CI	r^2^	r	*P*	95% CI	r^2^	r	*P*	95% CI	r^2^	r	*P*	95% CI	r^2^	r	*P*	95% CI
Kinovea vs Pro	0.867	0.931	0.347	− 0.236, 1.153	0.997	0.998	0.363	− 0.541, 2.570	0.912	0.955	0.010 *	0.306, 2.411	0.648	0.805	1.000	− 1.220, 0.970	0.994	0.997	1.000	− 3.585, 3.030
Kinovea vs Live	0.817	0.904	1.000	− 0.849, 0.699	0.888	0.942	1.000	− 7.787, 10.266	0.731	0.855	0.103	− 0.173, 2.556	0.717	0.847	1.000	− 1.161, 0.794	0.995	0.997	1.000	− 1.583, 4.145
Pro vs Live	0.606	0.778	0.901	− 1.639, 0.573	0.898	0.948	1.000	− 8.449, 8.899	0.761	0.872	1.000	− 1.886, 1.553	0.716	0.846	1.000	− 1.027, 0.910	0.993	0.996	1.000	− 1.986, 5.103
Vicon vs Kinovea	0.977	0.988	1.000	− 0.449, 0.199	0.985	0.992	1.000	− 3.933, 2.454	0.958	0.979	0.004 *	− 1.438, − 0.279	0.898	0.948	1.000	− 0.505, 0.705	0.999	0.999	1.000	− 2.024, 1.362
Vicon vs Pro	0.876	0.936	0.441	− 0.207, 0.874	0.989	0.994	1.000	− 2.581, 3.131	0.952	0.976	0.477	− 0.330, 1.330	0.847	0.920	1.000	− 0.631, 0.581	0.998	0.999	1.000	− 2.691, 1.475
Vicon vs Live	0.889	0.943	1.000	− 0.812, 0.412	0.952	0.976	1.000	− 5.390, 6.390	0.878	0.937	1.000	− 0.642, 1.309	0.915	0.957	1.000	− 0.630, 0.464	0.998	0.999	0.749	− 0.884, 2.784

Statistical significance level set to *P* = 0.05.

**Figure 2:**
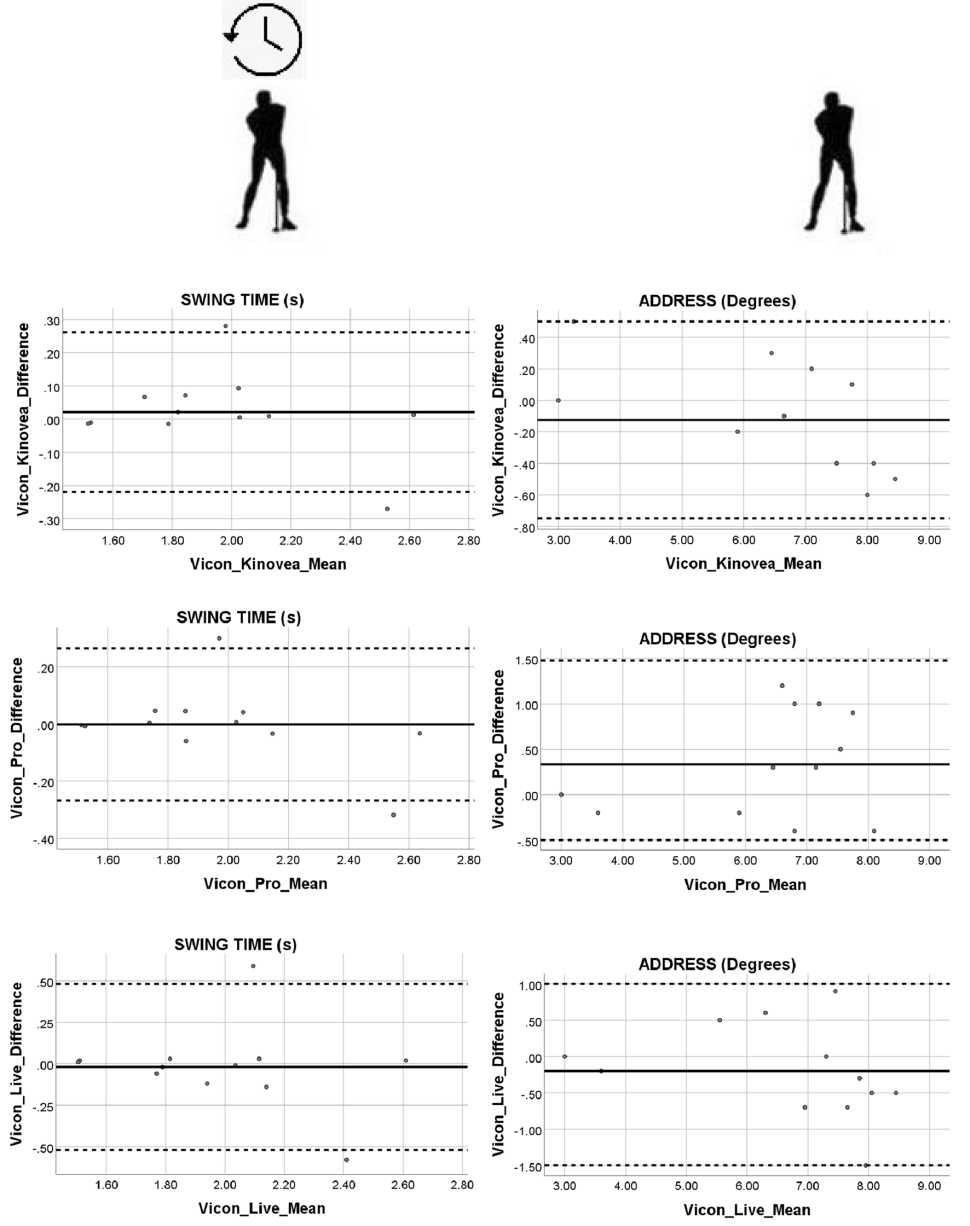
Swing time and address difference against mean for software data. Swing time was measured in seconds and address measured in joint angles (degrees).

The 2D and 3D motion analysis systems showed low levels of intra subject variability in all kinematic and temporal variables as indicated by the size of the standard deviations across the three trials. The results showed a high intra-rater reliability for both the kinematic and temporal parameters (ICC range: 0.929–0.999). With respect to swing time and golf phases, the ICC value for the intra-rater reliability test was 0.929 for the swing time and ranged from 0.963 to 0.999 for the kinematic golf phase variables. The CCA reliability statistics produced a value of 0.924 for the swing time and a range from 0.961 to 0.999 for the kinematic golf phase variables. The inter-rater reliability test for the swing time ranged from poor (0.471) to strong (0.995). All other kinematic golf phase variables were strong and ranged from 0.779 to 0.999. The swing time for the backswing and swing time for the downswing were similar across the 2D commercial software and 3D Vicon motion system. The ratio of the swing time for backswing to the swing time for downswing was 2:1. With respect to the software packages the swing time for the backswing and swing time for the downswing were 0.97 ± 0.21 s and 0.45 ± 0.12 s respectively. The swing time results (Fig. 2) from the Bland–Altman test showed two outliers located outside the 95% CI limits. The regression results for swing time between the 3D Vicon Motion System and the 2D commercial software produced unstandardized coefficient Beta values (B ≤ − 0.157) for the mean as being close to zero and not significant (*P* ≤ 0.318). The address showed a cluster of outputs where the mean was high (Fig. 2). The regression for address showed a similar trend with the unstandardized coefficient Beta values (B ≤ 0.120) for the mean being close to zero but with significance (*P* = 0.026) observed only for the 3D Vicon Motion System and the 2D Kinovea commercial software mean.

**Figure 3:**
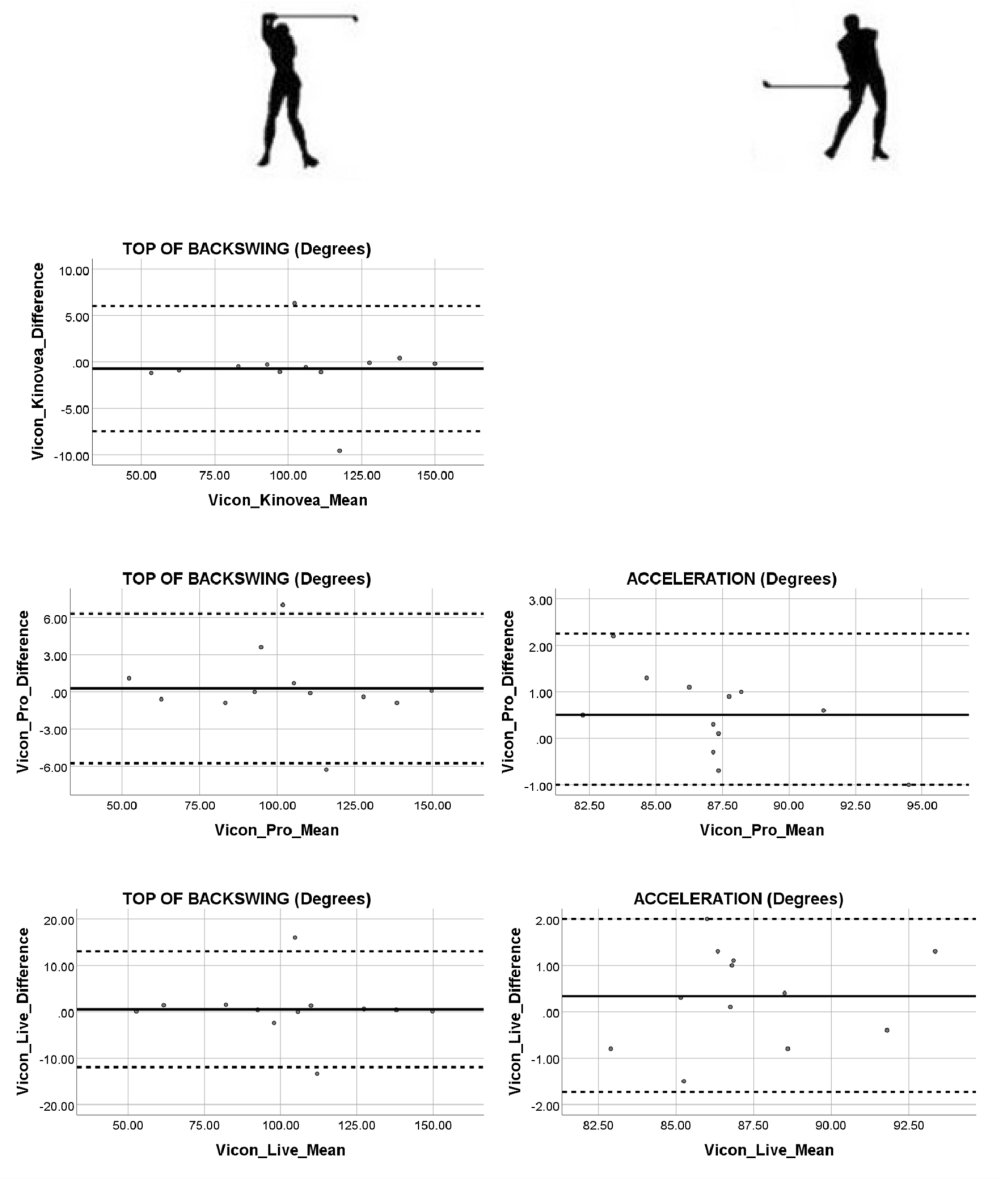
Top of backswing and acceleration difference against mean for software data. Both phase measurements are reported in joint angle (degree).

Two outliers located on either side of the upper and lower limits of the 95% CI limits were observed for all the top of the backswing Bland–Altman plots (Fig. 3). The regression results for the top of the backswing were similar with the unstandardized coefficient Beta values (B ≤ − 0.157) for the mean as being close to zero and not significant (*P* ≤ 0.922). With respect to the Bland–Altman test for the acceleration golf phase, the one sample t-test between the 3D Vicon Motion System and 2D Kinovea System difference produced a significant difference (P = 0.001) suggesting that we could not proceed with the Bland–Altman plot (Fig. 3). The other regression results for acceleration produced similar unstandardized coefficient Beta values (B ≤ − 0.162) for the mean as being close to zero and not significant (*P* ≤ 0.532).

**Figure 4:**
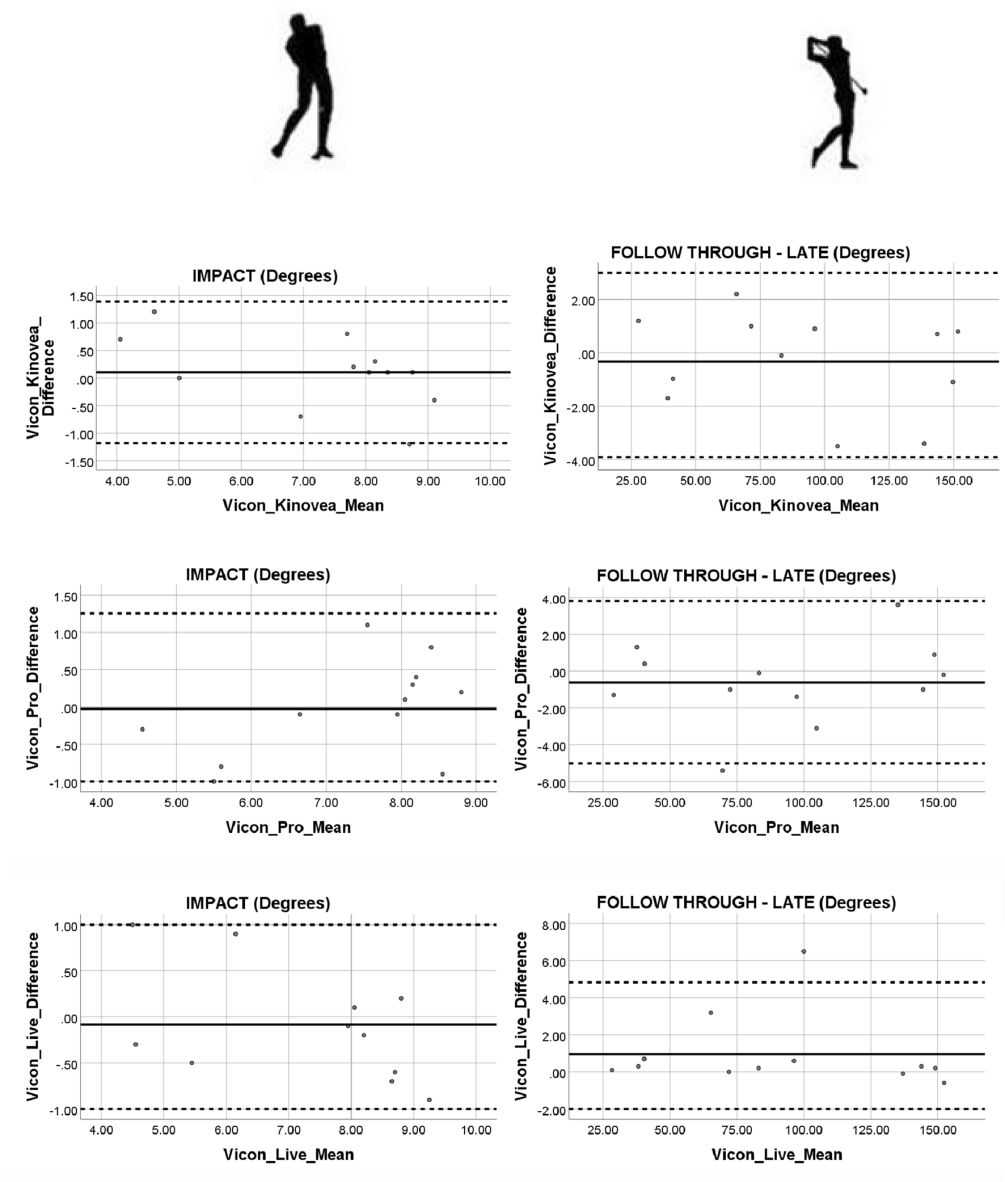
Impact and follow through (late) difference against mean for software data. Both phase measurements are reported in joint angle (degree).

Both regression results for the impact and follow through (Fig. 4) showed a similar trend with the unstandardized coefficient Beta values (B ≤ 0.231) for the mean being close to zero and not significant (*P* ≤ 0.737).

## Discussion

The purpose of this study was to evaluate and compare the gold standard golf club angle and swing time parameters during golf swings using three, 2D low cost, augmented video-based portable systems (Kinovea, SiliconCoach Pro, SiliconCoach Live). The findings of this study suggest that all three AVPS can be used to effectively calculate club angle parameters and swing time. The results agree with the swing time hypothesis and with the club angle output hypothesis for the golf phase measurements.

Although the study involved ‘novice’ participants, a large proportion of the acceleration golf phase measurements fell slightly under the 90° inclination angle measurement as indicated by the descriptive statistical outputs in Table 1. The results revealed no significant differences between the 2D commercial software and 3D Vicon motion system software. The r^2^ values for the pro vs live at address indeed were lower than the other comparisons, however the output interpretation was considered moderate. The variations between the trials for the pro and live were not as close as the other commercial software and Vicon Nexus software package comparisons. This is acknowledged by the size of the standard deviation. These differences we feel may be attributed to the resolution of the software or display resolution during the manual data processing using the software tools. The ratio of the swing time for the backswing and swing time for the downswing was 2:1. The swing time duration marginally varied across the different software because the frames representative of the phases varied by a frame or two across the various software packages. This means that identical frame numbers from one software were not replicated across the four software packages. Instead, each time a new software package was opened the frames at the start and end of the swings were inspected via effective visual examination of the video frames. Indeed, this is an important point as all raters reviewed each of the software independently by not taking previous recorded readings into consideration during the data processing stages of the outputs. Furthermore, the inter-rater reliability study provided a better approach to evaluate the utility and accuracy of the tools presented.

From the Bland–Altman plots it was apparent that majority of the outputs fell within the 95% CI. However, upon inspection of the unstandardized coefficient Beta values and the coefficient significance levels it was clear to see that the Beta values were close to zero as expected except for the golf address phase which produced a significant difference (*P* = 0.026) for the 3D Vicon Motion System and the 2D Kinovea commercial software mean. In general, all other temporal and kinematic golf phase measurements with respect to the 3D Vicon Motion System and the 2D commercial software mean revealed no proportional bias and good agreement in the results. Figure 3 showed one subfigure missing from the Bland–Altman plot of the Vicon_Kinovea_Difference versus the Vicon_Kinovea_Mean for the Acceleration phase of the golf swing. Before the difference was plotted against the mean in the Bland–Altman plot, we ran a one sample t test. In the software SPSS the test variable was selected as the Vicon_Kinovea_Difference and the test value was set to the default value zero. The results from the one sample t test produced a p value of 0.001 which suggests that this is a statistically significant result. This means that we could not proceed with the Bland–Altman plot. Hence the reason for not plotting the Difference vs Mean for the Vicon_Kinovea dataset.

To date, this is the first study that has used multiple low cost 2D AVPS to evaluate and compare the club angle parameters and the swing time of a golf swing. While there are no recent studies that show 2D motion analysis for a golf swing, Wright provides a detailed historical perspective and description of motion capture technologies and methodologies that have influenced golf biomechanics, club fitting, coaching and golf instruction^46^. Biomechanical analysis of the golf swing is widely acknowledged as being difficult to interpret due to the complexity of the swing, as it has a 3D motion, multi-planar sequence, which is performed at very high speeds^43^. Many software systems available allow for the evaluation of movement. These software systems vary in cost and can be delivered across multiple platforms (PC, Phone, Tablet), or via the server, web or client base. This study has taken a subset of these systems, Kinovea (free PC based application), SiliconCoach Pro (purchased PC based application) and SiliconCoach Live (purchased web-based application) and compared their outputs. The environment in which these applications may be used can differ, but the importance of the validity of the results remains the same. As a limitation, our sample size was small; however, this study has proven that the AVPS can produce a reliable and valid output with little financial and time overheads.

Unfortunately, performance variables such as club head speed, ball speed, ball position relative to stance width, wrist angle at various phases of the golf swing, club path, launch angle were not incorporated as outcome measures in this research study. These missing components were not measured as the protocol for both the 3D motion system and AVPS did not include a marker on the golf club head. Also due to the high speed of the ball post impact, the ball position, ball pathway and ball distance could not be clearly determined and thus would have affected the level of accuracy needed to extract the desired performance outputs. Studies are underway that involve higher shutter speeds. This will prevent streaking and make the digitization of the markers more reliable. Furthermore, to calculate these performance variables, during the experiment validation process, three independent systems would have had to be used namely a Voice Caddie Swing Launch Monitor (or Trackman™ III Golf Swing) together with a 2D high speed video camera and 3D motion system.

The analyses benefitted from the reliability statistical measurements i.e. the ICC and CCA analysis producing reliability results comparable to the clinical study done by Ugbolue et al.^1^. Future studies may choose to use only amateur golfers or perhaps professional golfers with a low handicap. Presently, a further study is underway that provides more outcome measures that focus on the golfer’s motion and motions of the golf club and golf ball. In this study all participants used the same golf club. This reduced the likelihood of variability across golf clubs. Questions pertaining to how different shaft deflections are investigated (i.e. What is used as a point of reference? How would a very flexible shaft be handled versus a very stiff?) are questions worthy of consideration for a future study. This may also show greater reliability and validity as their base technical model may display greater consistency across all swings. Although a detailed analyses has been performed while our study design compared within-player means, it is important to note that repeated measurements also provide useful information about the reliability of the measurement system (combined with human variability in this case as repeated swings are measured). Finally, the use of 2D AVPS software packages should be encouraged among movement analysis researchers and coaches, particularly when 3D motion capture systems and software cannot be accessed.

## Conclusion

Overall, the results from the three software packages were compatible as reflected by the descriptive statistics including the SEM and SM results. Further analyses revealed no significant differences for the ANOVA results with respect to the measured phases of the golf swing. High intra-rater reliability, CCA kinematic and temporal parameter measurements were obtained. The inter-rater reliability test for the swing time and kinematic golf phase measurements on average were strong. Aside from the address position, in general all temporal and kinematic outputs for the golf phase measurements with respect to the 2D commercial software and 3D Vicon motion software showed good agreement and no proportional bias in the results.

These results challenge the debate surrounding the purported errors and inaccuracies associated with the 2D AVPS. 2D AVPS are therefore useful, cost effective, easy to operate, reliable and indeed accurate when used correctly and in accordance with the recommended protocol instructions and guidelines. Given the outcome of our results, it is envisaged coaches, clinicians and biomechanics researchers will be reassured and encouraged to use the 2D AVPS where possible for their movement analysis assessments and evaluations.

## Data Availability

The datasets used and/or analysed during the current study are available from the corresponding author on reasonable request.
